# Advances in Click Chemistry for Single-Chain Nanoparticle Construction

**DOI:** 10.3390/molecules18033339

**Published:** 2013-03-14

**Authors:** Ana Sanchez-Sanchez, Irma Pérez-Baena, José A. Pomposo

**Affiliations:** 1 Centro de Fisica de Materiales (CSIC, UPV/EHU)-Materials Physics Center, Paseo Manuel de Lardizabal 5, E-20018 San Sebastian, Spain; E-Mails: ana_sanchez@ehu.es (A.S.-S.); irma_perez@ehu.es (I.P.-B.); 2 Centro de Fisica de Materiales (CSIC, UPV/EHU)-Materials Physics Center, Paseo Manuel de Lardizabal 5, E-20018 San Sebastian, Spain; 3 Departamento de Fisica de Materiales, Universidad del Pais Vasco (UPV/EHU), Apartado 1072, E-20080 San Sebastian, Spain; 4 IKERBASQUE — Basque Foundation for Science, Alameda Urquijo 36, E-48011 Bilbao, Spain

**Keywords:** click chemistry, controlled polymerization, folding/collapse, single-chain nanoparticles, nanomedicine, catalysis

## Abstract

Single-chain polymeric nanoparticles are artificial folded soft nano-objects of ultra-small size which have recently gained prominence in nanoscience and nanotechnology due to their exceptional and sometimes unique properties. This review focuses on the current state of the investigations of click chemistry techniques for highly-efficient single-chain nanoparticle construction. Additionally, recent progress achieved for the use of well-defined single-chain nanoparticles in some promising fields, such as nanomedicine and catalysis, is highlighted.

## 1. Introduction

**Figure 1:**
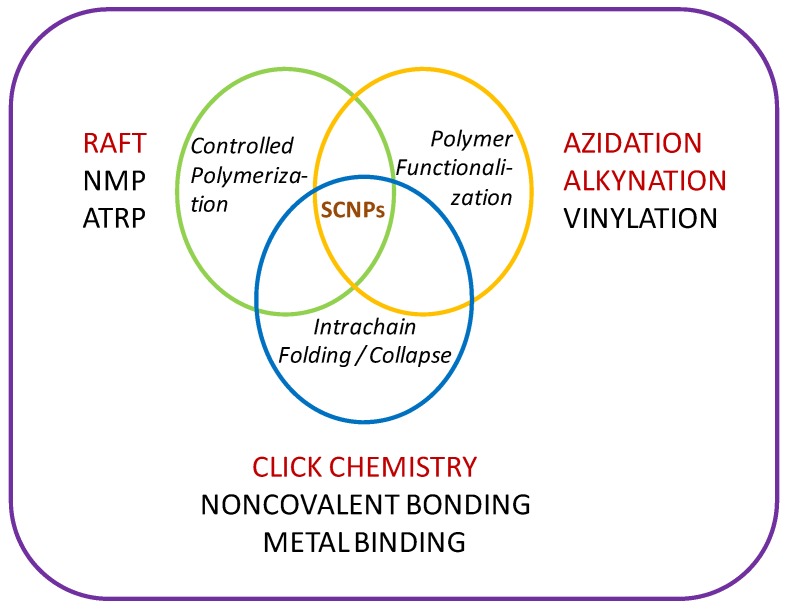
Illustration of the different techniques involved in the construction of single-chain nanoparticles (SCNPs): controlled polymerization, polymer functionalization and polymer folding/collapse. In red color are indicated highly-efficient procedures for SCNP construction (see Section 1.1. for acronyms).

Single-chain nanoparticles (SCNPs) are the smallest unimolecular nano-objects (size < 15 nm) that can be prepared starting from a linear polymer chain through *intrachain* folding/collapse [1]. It is worth mentioning that the controlled folding/collapse process during SCNP formation is reminiscent of protein folding to the native functional state, although single-chain nanoparticles mimic the globular structure of biomacromolecules only in a very rough, primitive manner. This is a consequence of the polydisperse nature (in size and composition) of current synthetic polymers showing a distribution of molar mass when compared to the perfectly monodisperse nature and precise chemical sequence of natural biomacromolecules. Even so, due to their exceptional properties, unimolecular polymeric nanoparticles have been evaluated as rheology agents [2,3], enzyme mimics [4,5], drug/siRNA/peptide nanocarriers [6,7,8,9], image contrast agents [3,10,11,12], sensors [13], catalytic systems in water [14], and smart gels [15]. As illustrated in Figure 1, synthesis of single-chain nanoparticles relies on the use of a combination of three main different techniques [3]. The first one is *controlled polymerization*, allowing the development of well-defined single-chain polymeric precursors of controlled molar mass and narrow size distribution to guarantee as much as possible the uniformity of the resulting unimolecular nanoparticles. Of the several controlled polymerization methods described in next section, reversible addition fragmentation chain transfer (RAFT) polymerization allows an exquisite control of the molecular weight distribution of the single-chain nanoparticle precursor and is a tolerant technique to monomers having a broad range of functional groups, without involving any metal catalyst. 

The second technique involved is *polymer functionalization*, allowing the decoration of the uniform polymeric precursor chains with appropriate functional groups for the corresponding folding step to collapsed single-chain nanoparticles. Under certain circumstances, the use of this second technique can be avoided through the preparation of well-defined copolymer precursors containing both inert and reactive functional groups distributed along the individual chains (often in a random, statistical fashion). A typical example of polymer functionalization step is the facile and quantitative transformation of the chloromethyl functional groups in poly(4-chloromethyl styrene-*co*-3-trimethylsilyl-propyn-1-yl methacrylate) copolymers to azidomethyl moieities *via* azidation with sodium azide [3]. 

The third fundamental technique involved is *intrachain folding/collapse* being used for the efficient transformation of the precursor coils to folded unimolecular nanoparticles. Obviously, when compared to the long evolution of proteins to their current functional status in living organisms, the field of single polymer folding to functional nanoparticles is still in its infancy. A nice example of highly-efficient folding/collapse method is certainly that based on intrachain click chemistry which has been used for robust, permanent single-chain nanoparticle construction *via* copper-catalyzed azide alkyne cycloaddition (CuAAC) [3].

### 1.1. Controlled Polymerization

**Figure 2:**
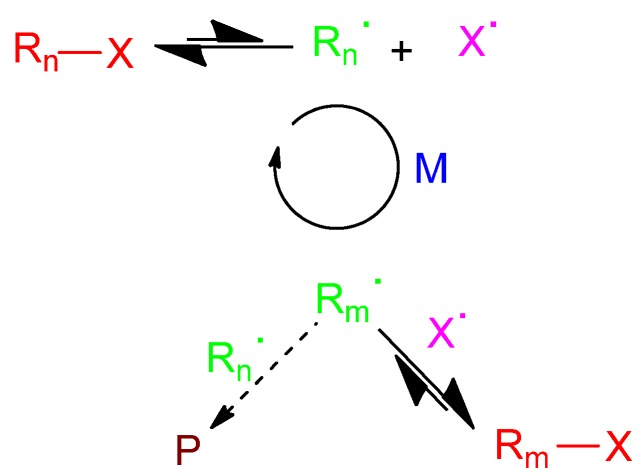
Equilibrium between dormant (R_n_-X, R_m_-X) and active (R_n_**^.^**, R_m_**^.^**) species in controlled radical polymerization (M: monomer; P: dead polymer by secondary reaction).

The development of controlled radical polymerization (CRP) processes allowing the facile construction of polymers approaching the polydispersity values of well-defined anionic “living” polymers with controlled architectures (stars, combs, dendrimers) and good tolerance towards the presence of a broad range of monomer functional groups has given rise to an exponential use of specialty polymers synthesized *via* CRP in nanoscience and nanotechnology applications [16]. Currently, the most common CRP techniques employed are reversible addition fragmentation chain transfer (RAFT) polymerization [17], atom transfer radical polymerization (ATRP) [18] and nitroxide mediated radical polymerization (NMP) [19]. A common feature of the variants of CRP is the existence of equilibrium between active free radicals and dormant, deactivated species. The exchange between active, growing radicals and dormant species allows the slow, but nearly simultaneous, growth of all chains (see Figure 2) while keeping the concentration of radicals low enough to minimize bimolecular termination reactions leading to dead polymer (P). 

Recently, ring opening metathesis polymerization (ROMP) using second generation of Grubbs catalysis has gained significant interest as a relatively new controlled polymerization technique [20]. A summary of the controlled polymerization techniques employed for the synthesis of single-chain nanoparticle precursors is provided in Table 1. I agree with the suggested changes, that can be made easily by the editorial office.

**Table 1:** Controlled polymerization techniques in the synthesis of SCNP precursors.

Polymerization Technique	Abbreviation	Nature of the SCNP Polymeric Precursors *
Reversible addition fragmentation chain transfer polymerization	RAFT	Poly(alkyl methacrylates) [5,6,7,10,15,21,22,23,24,25,26,27], poly(alkyl acrylates) [7,22,28,29], poly(styrene) [3,22,23,30,31,32], poly(haloalkyl styrene) [3,33], poly(4-N-Boc-vinylaniline) [33], poly(sodium 4-styrenesulfonate) [22], poly(N-alkyl acrylamide) [22,34,35]
Atom transfer radical polymerization	ATRP	Poly(alkyl methacrylates) [14], poly(alkyl acrylates) [11,12], poly(styrene) [36], poly(N-hydroxyethyl acrylamide) [37]
Nitroxide mediated radical polymerization	NMP	Poly(alkyl methacrylates) [9,38,39,40], poly(alkyl acrylates) [40], poly(styrene) [2,38,40], poly(haloalkyl styrene) [40], poly(fluorene) [41]
Ring opening metathesis polymerization	ROMP	Poly(ε-caprolactone) [38], poly(carbonates) [42] poly(norbornenes) [13,43,44]

* For SCNP copolymer and terpolymer precursors, only the nature of the main component is indicated.

### 1.2. Polymer Functionalization

Polymer functionalization aims at the quantitative and selective modification of a given polymer using relatively mild conditions without any side reactions. Polymer functionalization is also known as post-polymerization modification or polymer analogous modification and it has a long history in polymer science [45]. For natural polymers, the first report of sulfur-modified natural rubber was made independently about 1840 by Hancock, Ludersdorf and Goodyear [46]. Concerning synthetic polymers, the functionalization of butadiene polymers *via* thiol-ene addition was reported by Serniuk *et al.* [47] in 1948. Chlorinated polystyrene-divinylbenzene beads for ion exchange were developed by Pepper *et al.* [48] in 1953 and resins for solid-state peptide synthesis by Merrifield [49] on 1963. Systematic studies on the post-modification of epoxide-containing polymers were initiated by Iwakura *et al.* [50,51,52] in the early 1960s. The variety of chemical reactions available for post-polymerization modification increased significantly since the middle 1990s due to the emergence of controlled/living radical polymerization techniques (RAFT, ATRP, NMP) showing improved functional group tolerance when compared to classical living anionic or cationic polymerization processes. 

The most efficient and used polymer functionalization reactions are [45]: (1) thiol-ene/thiol-yne additions (click reactions), (2) modification of epoxides, anhydrides, oxazolines and isocyanates by reaction with amines/alcohols/thiols (click reactions), (3) modification of active esters by reaction with amines, (4) thiol-disulfide exchange, (5) Diels-Alder reaction (click reaction), (6) Michael-type addition, (7) Copper-catalyzed azide alkyne cycloaddition (CuAAC) (click reaction) and (8) Modification of ketones and aldehydes with amines / alkoxyamines / hydrazines. A summary of the different groups needed for the preparation of functionalized polymers *via* the above post-polymerization modification reactions is shown in Table 2. 

**Table 2:** Highly-efficient reactions available for the preparation of functionalized polymers *via* post-polymerization modification.

Polymer Functionalization Technique	Functional Groups Involved	Functionalizable Polymers
Thiol-ene / thiol-yne additions *	Thiol/alkene, alkyne	Polymers bearing alkene-, alkyne- or thiol-groups
Modification of epoxides, anhydrides, oxazolines and isocyanates by reaction with amines / alcohols / thiols *	Epoxide, anhydride, oxazoline, isocyanate/ amine, alcohol, thiol	Polymers containing epoxide-, anhydride-, oxazoline-, isocyanate-, amine-, alcohol- or thiol-groups [23]
Modification of active esters by reaction with amines	*N*-Hydroxysuccinimide, pentafluorophenyl ester/ amine	Polymers bearing *N*-hydroxy-succinimide-, pentafluorophenyl ester- or amine-groups
Thiol-disulfide exchange	Pyridyl disulfide/thiol	Polymers containing pyridyl disulfide- or thiol-groups [6]
Diels-Alder reaction *	Diene/alkene	Diene- or alkene-bearing polymers [28,40,53]
Michael-type addition	Acrylate, *N*-substituted-maleimide, vinyl sulfone/ thiols	Polymers bearing acrylate-, *N*-substituted-maleimide-, vinyl sulfone- or thiol-groups
Copper-catalyzed azide alkyne cycloaddition (CuAAC) *	Azide / alkyne	Azide- or alkyne-bearing polymers [3,10,21,22,30,34]
Modification of ketones and aldehydes with amines/ alkoxyamines/hydrazines	Ketone, aldehyde / amine, alkoxyamine, hydrazine	Polymers containing ketone-, aldehyde-, amine-, alkoxyamine- or hydrazine- groups

* Click chemistry techniques.

### 1.3. Intrachain Folding / Collapse

Notably, covalent bonding and non-covalent/dynamic-covalent bonding interactions have been used during controlled folding of linear polymeric precursors to permanent and reversible/stimuli-responsive single-chain nanoparticles as summarized in Table 3 and Table 4, respectively. 

**Table 3:** Covalent bonding interactions employed during SCNP construction for permanent polymer folding/collapse.

Reactive functional groups	Covalent bonding interactions
Vinyl [33,38,39,42]	Radical coupling & Cross-Metathesis
Benzocyclobutene [40,53]	Diels-Alder reaction *
Benzosulfone [9,28,41]	Diels-Alder reaction *
Azide + Protected alkyne [3,10,21,22,30,34]	Copper-catalyzed [3+2] cycloaddition **
Carboxilic acid [54]	Amide formation
Isocyanate [23]	Urea formation **
Enediyne [11,12,24,29]	Bergman & Photo-Bergman cyclization
Sulfonyl azide [31]	Nitrene-mediated cross-linking
Benzoxazine [36]	Ring opening polymerization
Alkyne [25]	Glaser-Hay coupling *

* C-C click chemistry. ** N-C click chemistry.

**Table 4:** Non-covalent (NC) and dynamic-covalent (DC) bonding interactions in SCNP construction.

Reactive functional groups	NC/DC bonding interactions
Benzamide [26]	Benzamide hydrogen bonding *
2-Ureido-Pyrimidone (UPy) [43]	UPy dimerization *
Coumarin [35]	Coumarin photo-dimerization **
Benzaldehyde [32]	Acylhydrazone formation **
β-Ketoester [27]	Enamine formation **
Methyl viologen + Naphtyl [37]	Cucurbit[*n*]uril complexation *
L-Phenylalanine (Phe) [55]	Hydrophobic Phe-Phe interactions *
Aminophenyl disulfide [44]	Disulfide formation **

* NC bonding interactions. ** DC bonding interactions.

In the present review article, we will focus on intrachain folding/collapse *via* click chemistry reactions leading to permanent single-chain nano-objects synthesized by starting from appropriate click chemistry precursors (see Table 3).

## 2. Single-Chain Nanoparticle Construction *via* Click Chemistry

The independent discovery in 2002 by Sharpless *et al.* [56] and Meldal *et al.* [57] that Huisgen 1,3-dipolar cycloaddition reactions between azides and alkynes can be carried out under very mild conditions and in a regioselective fashion when copper(I) is employed as catalyst inaugurated the *click chemistry* era. Nowadays, copper-catalyzed azide alkyne cycloaddition (CuAAC) has evolved into a common coupling technique in organic synthesis, polymer chemistry and materials science. In particular, the *click chemistry* concept [58] imposes specific requirements for a reaction to be classified as click chemistry such as: (1) to have a single-reaction trajectory, (2) to be chemoselective, (3) to be wide in scope (*i.e.*, applicable under a broad range of conditions with a multitude of starting substrates), (4) modular, (5) to give stable compounds, and (6) to show high yields. Additional requirements for reactions involving one or more polymeric reagents [59] to be classified as click reactions are: (7) to operate in fast time-scales, (8) to allow large-scale purification, and (9) to proceed with equimolarity. Three different methods have been developed for single-chain nanoparticle construction *via* click chemistry: (1) intrachain homocoupling, (2) intrachain heterocoupling, and (3) crosslinker-induced collapse.

### 2.1. Intrachain Homocoupling *via* Click Chemistry

**Figure 3:**
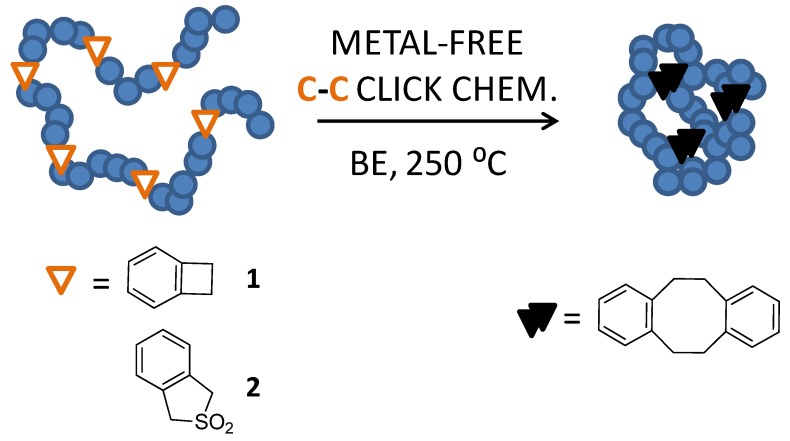
Illustration of single-chain nanoparticle construction through metal-free C-C click chemistry *via* Diels-Alder reaction in benzyl ether (BE) at high temperature (250 °C) involving benzocyclobutene (**1**) or benzosulfone (**2**) functional groups.

First use of a click chemistry reaction for single-chain nanoparticle construction was reported by Harth *et al.* [40] in 2002, relying on the use of a Diels-Alder-type reaction (*i.e.*, metal-free C-C click chemistry) involving benzocyclobutene (BCB) functional groups that were activated at very high temperature (250 °C, see Figure 3). The BCB group was selected by these authors due to its wide use as a latent Diels-Alder reagent in organic synthesis and in the formulation of thermosetting materials. Upon heating the BCB group above 200 °C, an extremely reactive *o*-quinoid structure was formed which reacted *via* an irreversible dimerization reaction to form a dibenzocyclooctadiene derivative as the major product. By using BCB -bearing polymer precursors, the direct result of the intrachain BCB homocoupling was the formation of intramolecular cross-linked single-chain nanoparticles under appropriate reaction conditions using a special continuous addition technique [40].

**Figure 4:**
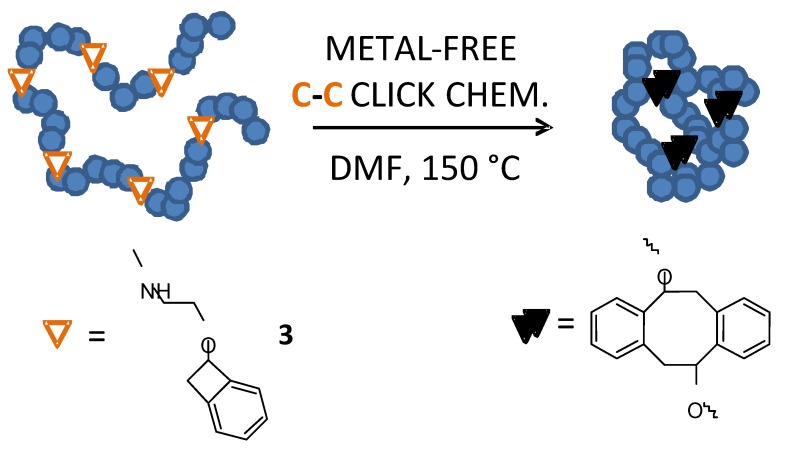
Single-chain nanoparticle formation through metal-free C-C click chemistry *via* Diels-Alder reaction in dimethyl formamide (DMF) at moderate temperature (150 °C) involving modified benzocyclobutene (**3**) functional groups.

The size of the resulting nanoparticles was accurately controlled by either the initial degree of polymerization of the polymeric precursor or the level of incorporation of BCB groups. Although the efficiency of this technique was certainly recognized, the synthesis at 250 °C in a high boiling point solvent (benzyl ether, BE) and its later removal were demanding procedures that limited their broad use. Further refinements of this technique were performed by Croce *et al.* [28] in 2007 by introducing benzosulfone reactive groups instead of BCB moieties (see Figure 3) and by Dobish *et al.* [53] in 2012 by developing substituted BCB functional groups reactive at lower temperatures (150 °C) (see Figure 4). 

**Figure 5:**
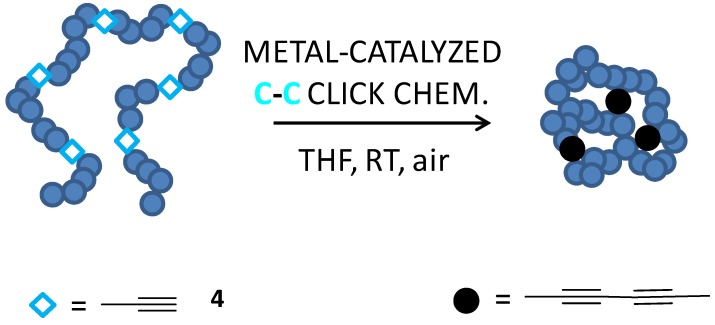
Synthesis of single-chain nanoparticles *via* Glaser-Hay coupling (metal-catalyzed C-C click chemistry) in tetrahydrofuran (THF) at RT under oxygen atmosphere involving unprotected alkyne (**4**) functional groups.

A very recent breakthrough in intrachain homocoupling for single-chain nanoparticle formation has been the use of Glaser-Hay coupling (*i.e.*, alkyne homocoupling) by Sanchez-Sanchez *et al.* [25] involving naked, self-clickable alkyne functional groups that were activated in a rapid and highly efficient manner at RT, under oxygen atmosphere, in tetrahydrofuran (see Figure 5). This metal-catalyzed C-C click chemistry technique was possible thanks to the successful synthesis *via* redox-initiated RAFT polymerization of single-chain nanoparticle precursors containing well-defined amounts of naked acetylenic functional groups available for intrachain metal-catalyzed C-C coupling. Accurate control over the molecular weight, polydispersity and composition of the linear poly(methyl methacrylate-*co*-propargyl acrylate), poly(MMA-*co*-PgA), precursor was demonstrated by working up to a maximum polymerization time of 20 h (*i.e.*, corresponding to a monomer conversion lower than 30%) and a maximum PgA monomer content in the feed of 35 mol%. 

### 2.2. Intrachain Heterocoupling *via* Click Chemistry

**Figure 6:**
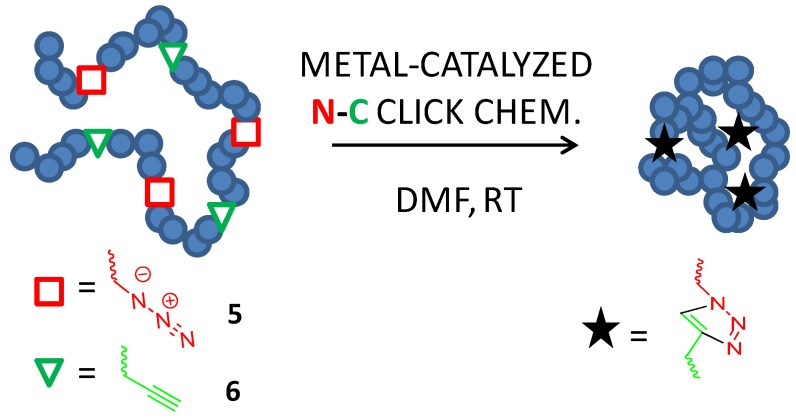
Illustration of single-chain nanoparticle construction through copper-catalyzed azide (**5**) alkyne (**6**) cycloaddition (CuAAC) (metal-catalyzed N-C click chemistry) in DMF at RT.

Copper-catalyzed azide alkyne cycloaddition, CuAAC (*i.e.*, metal-catalyzed N-C click chemistry), was used for the first time for the highly-efficient RT synthesis of bioconjugable poly(methyl methacrylate)-based single-chain nanoparticles by Ruiz de Luzuriaga *et al.* [21] in 2008 (see Figure 6). 

By using the intrachain heterocoupling method, bioconjugable polymeric nanoparticles were synthesized from poly(methyl methacrylate-*co*-3-azidopropyl methacrylate-*co*-3-trimethylsilyl-propyn-1-yl methacrylate) terpolymers synthesized by RAFT polymerization using a one-pot procedure and the continuous addition technique introduced by Harth *et al.* [40]. To illustrate the versatility of the technique, SCNPs containing an excess of azide groups were decorated with aminoacid moieties by performing a second CuAAC reaction in the presence of proparglyl glycine leading to amino acid-substituted single-chain nanoparticles. The method was further implemented by Oria *et al.* [3] by using a versatile click chemistry precursor of polystyrene single-chain nanoparticles involving poly(4-chloromethyl styrene-co-3-trimethylsilyl-propyn-1-yl methacrylate) copolymers that were transformed into poly(4-chloromethyl styrene-*co*-4-azidomethyl styrene-*co*-3-trimethylsilyl-propyn-1-yl methacrylate) terpolymers by a controlled and highly-efficiently azidation step with sodium azide in DMF. 

As a result, multifunctional polystyrene-based nanoparticles with diameters in solution of close to 4.2 nm were obtained showing improved rheology behavior (when mixed with elastomeric polymers) and useful fluorescence emission (when aromatic conjugation was maximized). More recently, this method has been employed by Ormategui *et al.* [34] to synthesize thermoresponsive poly(*N*-isopropyl acrylamide) single-chain nanoparticles with different intrachain cross-linking degree, showing a broad thermal coil-to-globule phase transition at neutral pH that was shifted to higher temperatures when compared to that of the SCNP precursor.

### 2.3. Crosslinker-Induced Collapse via Click Chemistry

**Figure 7:**
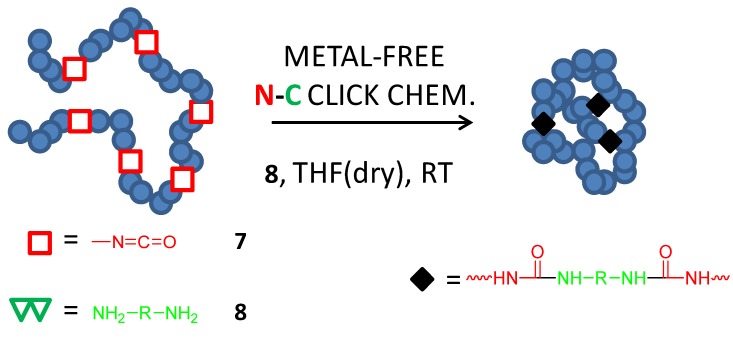
Synthesis of single-chain nanoparticles in dried THF at RT *via* metal-free N-C click chemistry involving intrachain bisurea cross-linking formation from isocyanate (**7**) groups and external diamine (**8**) cross-linker units.

Facile preparation of single-chain nanoparticles by working under strict absence of water *via* urea formation from isocyanates and diamines (metal-free N-C click chemistry) as schematically depicted in Figure 7 was demonstrated by Beck *et al.* [23] in 2009.

Nanoparticles with diameters ranging from 8 to 20 nm were synthesized with these materials showing physical properties (e.g., intrinsic viscosity, hydrodynamic radius) fully consistent with a collapsed, three-dimensional structure. More efficient and reproducible collapse was observed when the isocyanate copolymer was added to a solution of the diamine, by maintaing the total chain concentration below 0.1 mM. It was found that only about 75 mol% of the available isocyanate functional groups along the backbone underwent cross-linking with the remaining 25 mol% being available for further reaction with monofunctional amines. A similar volume reduction and cross-linking extend was observed for both styrenic and methacrylate based materials illustrating the efficiency and orthogonality of the urea-based intramolecular cross-linking reaction. Nanoparticle diameter was tuned either by controlling the molecular weight or the mole percentage of isocyanate functional groups.

**Figure 8:**
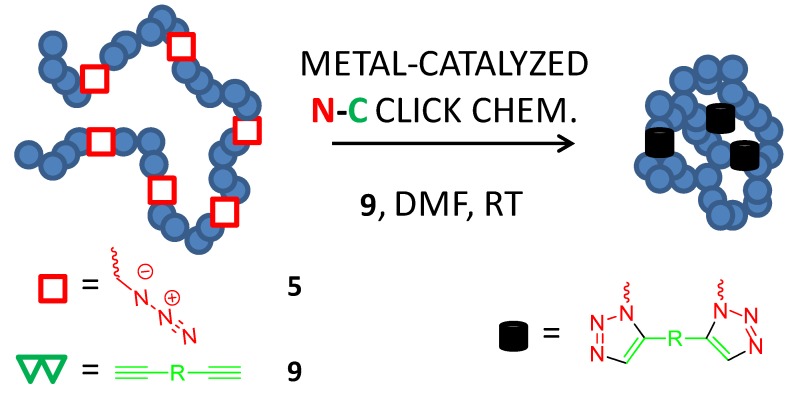
Illustration of single-chain nanoparticle construction in DMF at RT through CuAAC (metal-catalyzed N-C click chemistry) involving azide (**5**) functional groups and external dialkyne (**9**) cross-linking molecules.

The high versatility of the cross-linker induced collapse method *via* intrachain CuAAC was demonstrated by Ruiz de Luzuriaga *et al.* [22] in 2010 during the facile synthesis of single-chain nanoparticles of very different chemical nature (Figure 8).

With the assistance of this method, polystyrene, poly(alkyl (metha)acrylate), polymethacrylic acid, poly(sodium styrenesulfonate) and poly(*N*-isopropyl methacrylate) single-chain nanoparticles were synthesized in the 3–20 nm size range by starting with well-defined statistical copolymers with pending Cl- groups along the backbone that were transformed to N_3_- functional groups by treatment with NaN_3_ prior to cross-linker induced collapse. The generality of the method was illustrated by synthesizing single-chain nanoparticles of different chemical structure, solubility, functionality, physico-chemical properties and hence potential applications, covering from unconventional polyelectrolytes, melt rheology additives, drug delivery nanocarriers, fluorescent nanoprobes and magnetic resonance image agents [22]. 

Very recently, this method has been employed by Cengiz *et al.* [30] for the synthesis of polystyrene single-chain nanoparticles showing a 10-fold reduction in solution viscosity at 250 s^−1^ of shear rate when compared to the precursor polymer. This difference, that was even higher at very low shear rate, was attributed to the particle-like nature of the collapsed chains.

## 3. Applications of Single-Chain Nanoparticles

### 3.1. Nanomedicine

#### 3.1.1. Peptide/Drug/siRNA Controlled Delivery

Molecular dendritic transporter nanoparticle vectors based on hydrophilic single-chain nanoparticles post-modified *via* amide coupling reactions with dendritic molecular transport units (Newkome® dendrimers) were synthesized in a pionnering work by Hamilton *et al.* [9] in 2009 for the delivery of peptidic molecules into cells. The rapid transport of multiple copies of peptide units per particle across the cellular barrier into the cytoplasm of NIT 3T3 mouse fibroblast cells using this novel nanoscopic (5–10 nm in size) delivery system was demonstrated by confocal microscopy by using Alexa Fluor 568 dye as the label of the nanoparticle backbone and fluorescein as the marker of the peptide.

Facile hydrophobic guest (e.g., doxorubicin, Dox) encapsulation capabilities of biocompatible surface-functionalizable single-chain nanogels showing a diameter of 16 nm was demonstrated by Ryu *et al.* in 2010 [6]. Cell viability investigated by treating 293T human kidney cell lines with such nanogels showed high cell viability and no concentration-dependent toxicity, pointing to a nontoxic material for potential application in nanomedicine. 

Ultra-fine (7.4 nm in size) L-phenylalanine anilide-imprinted polymer single-chain nanoparticles have been synthesized by Njiang *et al.* [7] in 2012. Interestingly, the L-phenylalanine anilide-imprinted nanoparticles showed a higher sorption capacity for L-phenylalanine anilide (238 μmol/g) than for D-phenylalanine anilide (132 μmol/g) and the rate constant for L-phenylalanine anilide release was found to be inversely proportional to the squared radius of the particles.

As demonstrated by Tamura *et al.* [8] in 2009, nanosized (<100 nm) pH-sensitive polyamine nanogels containing poly(ethylene glycol) tethered chains are able to form spontaneously a polyion complex with negative charged siRNA through electrostatic interaction under physiological pH conditions. When combined a siRNA that knocks down the firefly luciferase gene, the nanogel/siRNA complex showed a remarkable enhancement of gene-silencing activity against firefly luciferase gene expressed in HuH-7 cells. Within certain confidence limits, similar results are expected for single-chain nanogels of lower size (<20 nm).

#### 3.1.2. Image Contrast Agents

Certain stable paramagnetic metal ion complexes, such as Gd^3+^ ones, enhance water proton relaxivity during image contrast in magnetic resonance imaging, MRI, of body tissues and fluids. Gd^3+^-containing nanoparticles were reported by Perez-Baena *et al.* [10] in 2010 by using a dialkyne crosslinker which was able to complex Gd^3+^ ions. The relaxivity value of the Gd^3+^-loaded single-chain nanoparticles on a per Gd basis was 6.78 mM^−1^ s^−1^, representing a modest 2-fold increase over a reference commercial low-molecular-weight Gd^3+^ chelate.

Very recently, water soluble single-chain nanoparticles containing ZnS nanocrystals (4.1 nm in size) have been reported by Zhu *et al.* [12] by using the single-chain nanoparticles as nanoreactors. Fast *in situ* growth of ZnS crystalline nuclei, leading to ZnS quantum dot formation was performed by treating directly Zn^2+^-containing single-chain nanoparticles with a sodium sulfite solution. The maximum photoluminescence intensity at 362 nm and the corresponding quantum yield were found to increase from 25 to 135 and from 2 to 17%, respectively, upon decreasing the size of the single-chain nanoparticles. Single-chain nanoparticles containing CdS quantum dots were also synthesized by these authors exhibiting bright fluorescence centered at 450 nm with a quantum yield of 45%.

As illustrated by Oria *et al.* [3] in 2010 poly(styrene)-based single-chain nanoparticles in which the intrachain cross-linking points consisted in triazole-benzene-triazole segments show a high level of fluorescence, that could be used for potential *in vivo* imaging applications. These single-chain nanoparticles present, after excitation at 350 nm, two maxima located at 391 and 407 nm respectively, in the fluorescence spectrum as well as a small shoulder at 424 nm. Control experiments in which the triazole-benzene-triazole conjugation was disturbed or absent failed to show this fluorescence pattern.

### 3.2. Catalysis

First report on the use of catalytic single-chain nanoparticles for carbonate hydrolysis was due to Wulff *et al.* [4]. Soluble single-molecule nanogels with molecular imprinted internal structure and containing just one active site per particle were reported by these authors in 2006. These single-chain nanoparticles showing 40 kDa in molecular weight were soluble in water/acetonitrile mixtures and displayed Michaelis-Menten kinetics in close analogy to natural enzymes, but with a very low turnover frequency (TOF) value of only 4.4 × 10^−3^ h^−1^. Recently, chiral nano-objects exhibiting catalytic activity towards carbonyl reductions in water without showing catalyst decomposition or hydrolysis have been reported by Terashima *et al.* [14]. These catalytic single-chain nanoparticles were prepared by folding through hydrogen-bonding interactions and helical self-assembly of a water-soluble amphiphilic precursor terpolymer containing both chiral and ruthenium-bonded units. The formation of a ruthenium-protecting hydrophobic compartment inside the single-chain nanoparticles was observed. Quantitative reduction of cyclohexanone to cyclohexanol in 18 h was demonstrated by using 0.5 mol% of supported Ru catalyst, corresponding to a turnover frequency of TOF = 11 h^−1^. Following the compartmentalization concept, Huerta *et al.* [5] have reported very recently a new family of single-chain nanoparticles which catalyze the aldol reaction in water with a turnover frequency of TOF = 125 h^−1^ by using only 0.5 mol% of supported L-proline organocatalyst. 

### 3.3. Other Uses

Additional use of single-chain nanoparticles for different applications has been proposed, such as rheology-improving agents for melts of thermoplastics [2], elastomeric polymers [3], nanocomposites [60] or paints [23,30], polyelectrolytes with unconventional behavior [22], compartmentalised sensors for metal ions [13], templates for the preparation of photoluminiscent carbon nanodots [12] and thermoresponsive hydrogels [15]. 

## 4. Conclusions

Different click chemistry methods have been implemented for efficient single-chain nanoparticle construction since the first one developed in 2002 based on Diels-Alder-type reactions taking place at 250 °C (metal-free C-C click chemistry). Recent refinement of this click technique allows one to carry out the synthesis at 150 °C that is still far from ideal RT conditions. 

Two significant breakthroughs in the field are worth mentioning: (1) the synthesis of single-chain nanoparticles at RT *via* intrachain CuAAC (metal-catalyzed N-C click chemistry) introduced in 2008, and (2) the construction of single-chain nanoparticles at RT under normal oxygen atmosphere *via* intrachain Glaser-Hay coupling (metal-catalyzed C-C click chemistry) developed in 2012. The requirement for a severe anhydrous reaction medium clearly limits the scope of the current version of metal-free N-C click chemistry *via* intrachain bisurea formation for single-chain nanoparticle preparation at RT. 

Undoubtedly, with the introduction/rediscovery of alternative click chemistry reactions (e.g., thiol/ene and thiol/yne additions) new advances in single-chain nanoparticle construction *via* intrachain click chemistry are expected to occur in the very near future, allowing the use of these versatile, artificial folded nano-objects of ultra-small size in a growing number of emerging applications.
